# Iron Deficiency Anemia as a Risk Factor for Osteoporosis in Taiwan: A Nationwide Population-Based Study

**DOI:** 10.3390/nu9060616

**Published:** 2017-06-16

**Authors:** Mei-Lien Pan, Li-Ru Chen, Hsiao-Mei Tsao, Kuo-Hu Chen

**Affiliations:** 1 Institute of Information Science, Academia Sinica, Taipei 115, Taiwan; mlpan66@gmail.com (M.-L.P.); hsiaomei.tsao@gmail.com (H.-M.T.); 2 Department of Physical Medicine and Rehabilitation, Mackay Memorial Hospital, Taipei 104, Taiwan; gracealex168@gmail.com; 3 Department of Mechanical Engineering, National Chiao-Tung University, Hsinchu 300, Taiwan; 4 Department of Obstetrics and Gynecology, Taipei Tzu-Chi Hospital, The Buddhist Tzu-Chi Medical Foundation, Taipei 231, Taiwan; 5 School of Medicine, Tzu-Chi University, Hualien 970, Taiwan

**Keywords:** iron deficiency anemia, osteoporosis, ferrum infusion, blood transfusion

## Abstract

The cause-effect relationship between iron deficiency anemia (IDA) and osteoporosis has not been established in the general population. Thus, the current longitudinal study determined the role of IDA as a risk factor for osteoporosis by analyzing a large nationwide population-based sample. In a sample of 1,000,000 randomly sampled individuals from the 1998–2012. Taiwan National Health Insurance Research Database, patients with IDA (case group (*n* = 35,751)) and individuals without IDA (control group (*n* = 178,755)) were compared. Patients who were <20 years of age and who had pre-existing osteoporosis prior to the diagnosis of IDA were excluded. Each patient with IDA was age- and gender-matched to five individuals without IDA. The diagnoses of IDA and osteoporosis (coded using ICD-9CM) were further confirmed with blood test results and X-ray bone densitometry to ensure the accuracy of the diagnoses. Osteoporosis occurred more often among patients with IDA compared to individuals without IDA (2.27% vs. 1.32%, *p* < 0.001). Cox proportional hazard analysis revealed that the risk for osteoporosis was significantly higher in the case than the control group (hazard ratio (HR) = 1.74; 95% CI = 1.61–1.88) and remained similar after adjustment for covariates (adjusted HR = 1.81; 95% CI = 1.67–1.97). Compared with individuals without IDA, the risk for osteoporosis was even higher for patients with IDA who received intravenous ferrum therapy (adjusted HR = 2.21; 95% CI = 1.85–2.63). In contrast, the risk for osteoporosis was reduced for patients with IDA who received a blood transfusion (adjusted HR = 1.47; 95% CI = 1.20–1.80). As a predictor, prior IDA is a significant and independent risk factor for development of osteoporosis.

## 1. Introduction

Osteoporosis, characterized by a systemic impairment of bone mass, strength, and microarchitecture that results in fragility fractures [1], is a major health issue worldwide. Defined as low bone mineral density (BMD) with a *T* score < −2.5 [2], osteoporosis is common in aging populations and affects 40% of menopausal women [1], among whom the cumulative fracture lifetime risk is as high as 60% [3,4]. Because bone loss occurs insidiously and is initially asymptomatic, and osteoporosis is often only diagnosed after the first clinical fracture has occurred, early assessment of an individual’s risk for osteoporosis is therefore important in an effort to prevent the first fracture [1]. According to our report [3] and others [5], maintaining a calcium intake of at least 1000–1200 mg/day has long been recommended for individuals to treat and prevent osteoporosis.

Anemia is another important global health problem and common medical condition occurring in daily clinical practice [6]. Microcytic anemia, the most prevalent type of anemia, is characterized by the production of red cells that are smaller than normal. The causes of microcytic anemia can be classified into a lack of globin product (thalassemia), restricted iron delivery to the heme group of hemoglobin (anemia of inflammation), a lack of iron delivery to the heme group (iron-deficiency anemia (IDA)), and defects in the synthesis of the heme group (sideroblastic anemia) [7]. The human body has evolved to conserve iron in several ways, including iron recycling after the breakdown of red cells and iron retention in the absence of an excretion mechanism [6]. Because excess levels of iron can be toxic, the absorption of iron is limited to 1–2 mg daily, and most of the iron needed daily (approximately 25 mg/day) is provided through recycling by macrophages that phagocytose senescent erythrocytes. The latter two mechanisms are controlled by the hormone, hepcidin, to maintain total body iron homeostasis, thus avoiding iron deficiency and iron excess [6]. In patients with variant causes of IDA, including malnutrition, infections, heavy menstrual loss, or blood loss from the gastrointestinal tract, iron therapy or blood transfusion is often considered to correct the problem [6,7].

The main mechanism underlying osteopenia and osteoporosis involves abnormal bone remodeling; an imbalance in bone turnover between osteoclasts, which creates an excessively deep cavity (resorption); and osteoblasts, which refill the normal resorption cavity (formation) [8,9]. By inhibiting the Wnt signaling pathway, sclerostin, a non-classical bone morphogenetic protein (BMP) antagonist, has been identified as a negative regulator of bone formation, and its effect can be inhibited by using strontium ranelate [10]. Common risk factors for osteoporosis include a personal history of fracture, current low bone mass, or body mass index (BMI), advanced age, female gender, menopausal status, low lifetime calcium intake, vitamin D deficiency, an inactive lifestyle or extended bed rest, and use of corticosteroids [1]. Although it has been hypothesized that severe iron deficiency is associated with osteoporosis [11,12], iron overload is well-recognized as a risk factor for osteoporosis [9,13,14,15]. Currently, there is a paucity of research exploring the association of thalassemia [9,15,16] or pernicious anemia [17] with osteoporosis; however, the relationship between IDA, the leading cause of anemia [6,7], and subsequent osteoporosis remains unclear. Although some studies have demonstrated that anemia is associated with osteoporosis [18,19,20], the cause–effect relationship cannot be established [21] due to the limitation of a cross-sectional study design in all of the studies. Moreover, the generalization or application of conclusions in these three studies was largely limited because all of the participants were elders, the number of participants in all studies was not large (<1000), and these studies used non-standard measurement of BMD, such as ultrasound or CT scan of the calf rather than standard X-ray bone densitometry (DEXA) for the diagnosis of osteoporosis [18,19,20]. The cause–effect relationship between IDA and osteoporosis has not been clarified in the general population. Thus, the current study explored the role of IDA as a risk factor for osteoporosis by analyzing a large nationwide population-based sample with stricter selection criteria and longitudinal follow-up.

## 2. Materials and Methods

### 2.1. National Health Insurance Research Database

The National Health Insurance (NHI) program in Taiwan is a single-payer, compulsory health insurance program. After its launch in 1995, the enrollment rate increased gradually from 93% to 99% by 2010. For research purposes, the claims data were de-identified by scrambling the identification codes of patients and medical facilities to constitute Taiwan’s National Health Insurance Research Database (NHIRD). This database includes the reimbursement records of ambulatory care and hospitalization—such as demographic data, dates of clinical visits, disease diagnoses, examinations, treatment procedures, and pharmacy dispensing—and uses the International Classification of Diseases, Ninth Revision, Clinical Modification (ICD-9-CM) for diagnostic coding. The Longitudinal Health Insurance Database 2010 (LHID2010), a subset of NHIRD data, represents 1,000,000 randomly sampled beneficiaries insured in 2010 from the NHI. This dataset has been confirmed to have no significant difference in age, gender, or healthcare costs from the entire population, which consists of all beneficiaries under the NHI program. We conducted a retrospective cohort study to estimate the risk of subsequent osteoporosis in patients with IDA using the LHID2010. The study was conducted in accordance with the Declaration of Helsinki, and the protocol was reviewed by the Institutional Review Board of Taipei Tzu-Chi Hospital, Taiwan (approval number: 03-XD15-040).

### 2.2. Study Population and Study Design

We identified patients diagnosed with IDA between 1998 and 2012 from LHID2010. IDA was defined and coded as ICD-9-CM code 280.X combined with complete blood count (CBC) and ferritin tests. According to the World Health Organization (WHO) criteria, anemia is defined as a hemoglobin level <13 g/dL for males and <12 g/dL for females [22]. IDA is further specified by a mean corpuscular volume (MCV) <80 and a serum ferritin level <15–30 µg/L [6,23]. Individuals who had missing data, and those who were diagnosed without blood tests were excluded. The date of initial IDA diagnosis was set as the index date. In the current study, we focused on IDA. Thus, the patients <20 years of age were excluded to eliminate the potential confounding effect because they may have had congenital anemia or anemia caused by other diseases. To clarify the cause-effect relationship between IDA and osteoporosis, those patients who were diagnosed with osteoporosis before anemia were also excluded. Osteoporosis was confirmed with a coding of ICD-9-CM code 733.0X, and defined as a *T* score < 2.5 standard deviations under X-ray bone densitometry [1,2]. A total of 35,751 individuals diagnosed with IDA comprised the case group. After excluding individuals with any records of anemia, the remaining individuals in the LHID2010 were considered to be candidates for the control group. We then matched five individuals without IDA (the control group) to each individual diagnosed with IDA (the case group) by age and gender. Since the index date, all individuals with IDA and the matched controls were followed until they developed osteoporosis or until 31 December 2012, whichever occurred first.

### 2.3. Covariates

The general characteristics of individuals analyzed in this study included age at the time of IDA and osteoporosis diagnosis, gender, occupation, the degree of urbanization, insurable salary, and co-morbidities. The occupation type consisted of blue collar, white collar, and retired/others. The degree of urbanization was categorized into three levels (urban, suburban, and rural). The insurable salary was divided into a monthly insurable wage <NTD 20,000, NTD 20,001–40,000, ≥NTD 40,000, and others (spouse/dependents). The current exchange rate is US $0.0316 = NTD1.00. The co-morbidities consisted of common medical diseases and disorders that may possibly affect anemia or osteoporosis. Common diseases included hypertension (401.X–405.X), diabetes mellitus (250.X), dyslipidemia (272.X), congestive heart failure (428.0), coronary artery disease (414.0X), cerebrovascular disease (430.X–438.X), and chronic pulmonary disease (490.X–496.X). Disorders that may possibly affect anemia or osteoporosis included end-stage renal disease (585.X), disorders of the thyroid gland (240.X–242.X), parathyroid glands (252.X), and adrenal glands (255.X), disorders of menstruation (626.X), menopause (627.X), digestive ulcer or hemorrhage (530.X–534.X), liver disease (571.2X, 571.5X, 571.6X), and a history of previous fractures (733.1X and 733.8X).

### 2.4. Statistical Analysis

For analysis of general characteristics of the individuals in the case and control groups, categorical and continuous variables were analyzed using chi-square and *t*-tests, respectively. To assess the risk of developing subsequent osteoporosis, we performed Cox’s regression analysis to obtain the crude and adjusted hazards ratios (HRs) and 95% confidence intervals (CIs) for the case group compared with the control group. Cox’s regression models were adjusted for age, gender, occupation, the degree of urbanization, insurance salary, and co-morbidities. Further, we calculated the HRs for the patients with IDA according to gender and treatments for IDA (without any treatment or use of oral iron only, use of intravenous ferrum, and blood transfusion). All data analyses were performed with SAS^®^ (version 9.4; SAS Institute, Inc., Cary, NC, USA). Statistical significance was set at a *p*-value < 0.05.

## 3. Results

**Figure 1:**
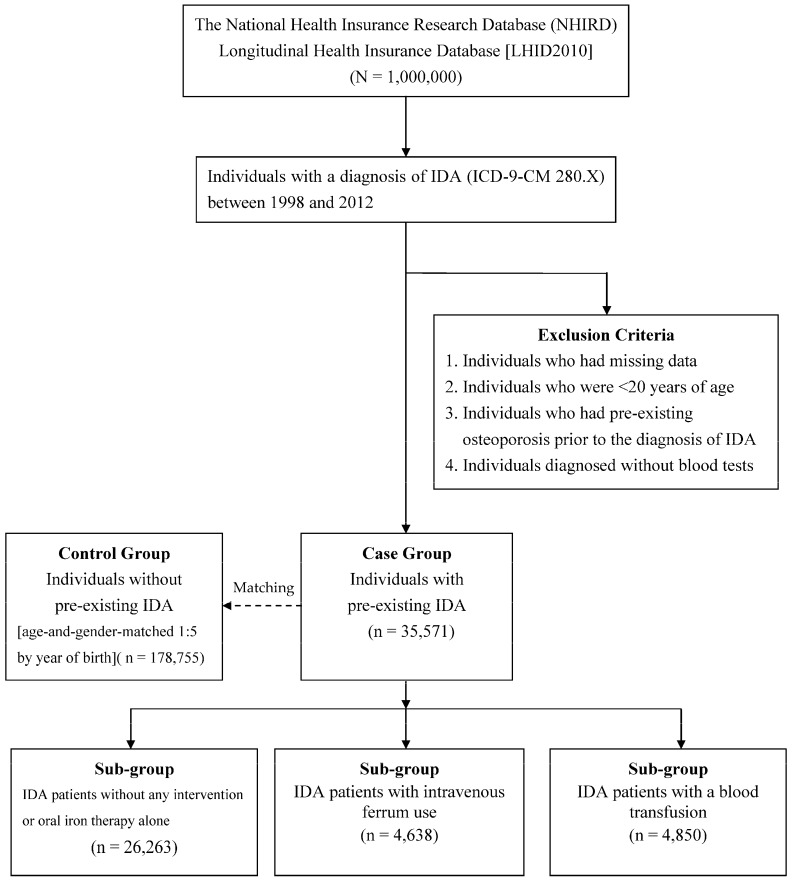
Flow chart of case inclusion, exclusion, and classification.

In the current study, a sample of 1,000,000 randomly sampled individuals was obtained from the national database LHID2012. According to the inclusion and exclusion criteria, 35,751 eligible individuals with a valid IDA diagnosis (case group) were identified, and we identified 178,755 individuals without IDA (control group; Figure 1). IDA was more prevalent in females than males (81.2% vs. 18.8%). The mean age at the time of diagnosis of IDA was 43.7 years. In comparison to individuals in the control group, patients in the case group developed subsequent osteoporosis at a younger age (59.1 vs. 62.5 years; *p*-value < 0.001). The occupation, degree of urbanization, and insurable salary were significantly different between the case and control groups, (all *p*-value < 0.001). In addition, the cases had higher rates of co-morbidities than the controls (all *p*-value < 0.001). The general characteristics of individuals in the case and control groups are shown in Table 1.

The incidence of subsequent osteoporosis was significantly higher in individuals in the case group than the control group (HR, 1.74; 95% CI, 1.61–1.88). After adjustment for age, gender, occupation, the degree of urbanization, insurable salary, and co-morbidities, the risk of developing osteoporosis remained similar (HR, 1.81; 95% CI, 1.67–1.97; Table 2). For the patients diagnosed with IDA, the average duration of IDA was 6.86 years. Additionally, for the patients who were finally diagnosed with osteoporosis in both groups, the time intervals from the index date of the case (age at IDA diagnosis) and control (age and gender matched) groups to the onset of osteoporosis were 4.17 and 4.81 years, respectively. The result implied that the individuals diagnosed with IDA had not only a higher rate but also an earlier onset of osteoporosis compared to those without IDA. Among patients with IDA, the number of the female patients (*n* = 29,017) was greater than the male patients (*n* = 6734), and the female patients had a higher ratio of subsequent osteoporosis than the male patients (2.55% vs. 1.07%). Additionally, both male and female patients with IDA had an increased risk for subsequent osteoporosis when compared to the corresponding controls (males: crude HR, 2.08; 95% CI, 1.58–2.73; adjusted HR, 1.99; 95% CI, 1.50–2.65 and females: crude HR, 1.71; 95% CI, 1.57–1.86; adjusted HR, 1.80; 95% CI, 1.65–1.96). It is worth noting that the risk for osteoporosis was slightly higher rather than lower in females after adjustment for covariates. The risks for subsequent osteoporosis in patients diagnosed with IDA, stratified by gender, are shown in Table 3. For the female patients, the types and rates of co-morbidities were significantly different from male patients, among whom there were no menstruation disorders or menopause (Table 1).

In contrast to individuals without IDA (control group; *n* = 178,755), 26,263 patients with IDA had not undergone any intervention or had used oral iron therapy only. Among patients with IDA, there were 4638 who received intravenous ferrum therapy, and 4850 who received a blood transfusion. Further analyses stratified by use of intravenous ferrum and blood transfusion showed that the risk for subsequent osteoporosis was elevated when patients with anemia were treated with intravenous ferrum therapy (adjusted HR, 2.21; 95% CI, 1.85–2.63) compared to patients with anemia who had not undergone any intervention or who used oral iron therapy only (adjusted HR, 1.80; 95% CI, 1.64–1.98); however, the risk for subsequent osteoporosis was decreased when patients with anemia were treated with a blood transfusion (adjusted HR, 1.47; 95% CI, 1.20–1.80; Table 4).

## 4. Discussion

The results of the current study revealed that patients with a history of IDA had a higher incidence of osteoporosis. Compared with individuals without IDA, patients with a history of IDA had a near two-fold risk for osteoporosis. Appropriate information and suggestions should be provided to people at-risk after confirmation of IDA to facilitate earlier medical management or specialty referral. Counseling and management can be considered for affected patients to improve symptoms resulting from osteoporosis, such as backache and joint pain, and to prevent known complications, such as fractures. Overt anemia can be corrected by giving blood transfusion therapy. On the other hand, the use of intravenous ferrum for IDA may be associated with a higher risk for osteoporosis.

Our results showed that in the national population, the number of female patients (*n* = 29,017) was greater than the male patients (*n* = 6734) among patients with IDA. For women of reproductive age, the reason for the finding is presumably periodic blood loss during menstruation. Another possible reason is that, compared to men without periodic blood loss, women with hypermenorrhea have more frequent visits to clinics or hospitals, where the ICD codes are coded and the diagnosis is obtained accordingly. As expected, female patients had a higher ratio of subsequent osteoporosis than the male patients (2.55% vs. 1.07%). Indeed, women would experience menopause because of decreased estrogen production, and thereafter there is no further protective effect of estrogen against osteoporosis.

Well-known causes of IDA include malnutrition, infections, vegetarian diets, and acute or chronic blood loss due to heavy menstruation, GI tract diseases, or other etiologies [6]. The pathways between pre-existing IDA and osteoporosis are not fully understood. Currently, it is hypothesized that blood loss intensifies hematopoiesis by increasing the level of hematopoietic growth factors with subsequent proliferation of hematopoietic cells, including osteoclasts, while at the same time stimulating proliferation of osteogenic progenitor cells with a subsequent increase in osteoblasts [24]. Because activation of hematopoietic growth factors is greater than proliferation of osteogenic progenitor cells, resorption of bone tissue, and extension of hematopoietic territories will eventually result in osteoporosis [24]. This hypothesis was supported by another animal study that showed augmented production of the hematopoietic microenvironment, a relative reduction in the amount of generated bone, and activation of the bone resorptive process in the newly forming osteohematopoietic complex in mice with chronic blood loss and anemia [25]. Another possible explanation for our finding is the simultaneous loss of blood calcium resulting from acute or chronic blood loss due to heavy menstruation or GI tract diseases. Furthermore, a part of IDA is attributed to malnutrition, which suggests sporadic deficiencies in elements and nutrients, including iron and calcium. All of the aforementioned conditions end in a reduction of calcium sources, and may play a critical role in the formation of subsequent osteoporosis. A detailed discussion of the underlying mechanism is beyond the scope of the current study, but we believe that further efforts can be made to explore the etiology that underlies osteoporosis in patients with pre-existing IDA.

Compared with no intervention, oral iron therapy alone, or intravenous ferrum use, blood transfusion request may indicate a more severe form of IDA. It is therefore reasonable to use different therapies for IDA as indicators to classify the severity of individuals with IDA. Accordingly, an available analysis that has been done in the current study was shown in Table 4, which classified IDA patients according to different therapies (without any intervention or with oral iron therapy alone, with intravenous ferrum use, with blood transfusion therapy). In fact, different therapies required for IDA patients implied different severity of anemia among these patients. It seemed that patients with more severe anemia had a higher risk of developing osteoporosis. As shown in Table 4, the risk of future osteoporosis for IDA patients receiving intravenous ferrum (adjusted HR, 2.21) was higher compared to those without any intervention or with oral iron therapy alone (adjusted HR, 1.80). Interestingly, the risk was lower for IDA patients receiving blood transfusion therapy (adjusted HR, 1.47). We supposed the reason was that blood transfusion therapy possibly restored blood calcium, which may be decreased during acute or chronic blood loss in some anemia patients. Possibly because of shortening duration of severe anemia, blood transfusion therapy was associated with a lower risk of osteoporosis for individuals with IDA. However, this is not the case in patients with IDA who underwent intravenous ferrum therapy. For these individuals, the risk (adjusted HR, 2.21) for osteoporosis was even higher than all individuals with IDA (adjusted HR, 1.81). The result is in agreement with the conclusions of previous studies that revealed iron load is a risk factor for osteoporosis [9,13,14,15]. Intravenous ferrum therapy is commonly used in patients with IDA; however, iron overload has been shown to affect bone remodeling directly by inhibiting the activity of osteoblasts [9,13]. Another report confirmed the role of iron excess in osteoporosis by inducing an oxidative stress reaction [14]. Ferrum infusion therapy has been noted to increase reactive oxygen species, and elevate serum TNF-α and IL-6 concentrations, thus subsequently inducing a dose-dependent increase in tissue iron content, changes in bone composition, and trabecular and cortical thinning of bone accompanied by increased bone resorption [14]. There was also evidence demonstrating that, in patients undergoing ferrum infusion therapy, iron deposition in the bone impairs osteoid maturation and inhibits mineralization. The mechanism involves the incorporation of iron into crystals of calcium hydroxyapatite, which consequently affect the growth of hydroxyapatite crystals [9,15]. On the other hand, iron overload inhibits osteoblast and fibroblast proliferation and differentiation, and collagen formation, while enhancing osteoclast apoptosis [9,15]. Another minor explanation we suggest is that ferrum-sole infusion therapy may have a “hemodilution effect” for blood calcium. Without transfusion of blood content, the blood calcium may be substantially decreased under ferrum infusion therapy alone, thus resulting in osteoporosis. The true mechanism warrants further investigation. For patients undergoing intravenous ferrum therapy to correct anemia, essential blood calcium supplementation may be considered at the same time to ensure at least adequate calcium sources in the circulation to ameliorate the effect of intravenous ferrum therapy on osteoporosis.

The current study focused on the role of iron deficiency anemia (IDA) as a risk factor for osteoporosis. Although other types of anemias were not the major concern of the current study, we have analyzed the association of osteoporosis with other types of anemias including pernicious or nutritional (Vitamin B12; folate) deficiency anemia (ICD 9 code 281.X), hereditary hemolytic anemia (thalassemias and sickle-cell anemia) (ICD 9 code 282.X), aplastic anemia (ICD 9 code 284.X), and other unspecified anemia (ICD 9 code 285.X). Between individuals with and without other types of anemias, the risks of developing osteoporosis were 2.68% vs. 1.65%, 1.46% vs. 0.73%, 2.83% vs. 1.10%, and 2.14% vs. 1.22%, respectively. The HRs (95% CIs) were 1.65 (1.42–1.92), 2.01 (1.60–2.54), 2.76 (1.80–4.23), and 1.78 (1.68–1.89), respectively (all *p* < 0.001). For all of the individuals with other types of anemias, the risk of developing osteoporosis was significantly higher compared with those without anemia.

To the best of our knowledge, this is the first large-scale study to explore the role of IDA as a risk factor for osteoporosis among the general population. Our study had several strengths, including a large sample size, sound sampling method, and stricter selection criteria for patients diagnosed with IDA or osteoporosis. One advantage of our study was that the sample was retrieved from the database of a general survey involving a national population rather than purposive sampling or special age (elderly) or gender (female) groups [18,19,20]. The sample size of the current study was large among all studies of its kind to investigate the incidence of osteoporosis for patients with anemia. Therefore, our results are robust due to minimization of possible errors that originate from the sampling process. Moreover, all diagnoses for patients with IDA or osteoporosis were established by confirmation of objective examinations and laboratory tests rather than by the subjective judgment of a physician. All biases resulting from the samples, the selection, the investigator, and the measuring process were minimized by the methods and criteria used. Additionally, we analyzed the effects of blood transfusion and intravenous ferrum therapy used for correcting anemia on subsequent osteoporosis, which has not been previously reported [18,19,20].

The current study has several inherent limitations despite its strengths. First, it is not easy to precisely define a medical condition from administrative data. Using ICD codes alone does not entirely reflect the real status of clinical conditions. Therefore, we not only used ICD codes to identify cases, but also considered reports of laboratory testing and instrument examinations as essential inclusion criteria to increase the appropriateness of the case definition. Second, under the NHI system, administrative data are collected for the purpose of reimbursement. Thus, the existing data could have been inconsistently collected over time. This inconsistency could have affected the results to some extent. Moreover, some demographic characteristics are lacking in the NHIRD, such as family history of fractures, body mass index (BMI), hemoglobin level, food intake, smoking habits, social status, and marital status. Thus, we were unable to investigate the contributing influence of these factors. Because the height and weight of every insured resident are not routinely recorded in this national health database of Taiwan, to obtain such data is not possible. Furthermore, although the power of the analysis of sub-grouping was adequate (>4000 individuals in each sub-group), we could not obtain the information on intravenous ferrum dosage and duration, which was a limitation of the national database and could have affected the study results. Due to the ethnic homogeneity of Taiwan, the results do not reflect the effect of the ethnicity on the association between IDA and osteoporosis. Consequently, the results might not be generalized to other ethnic populations. In this longitudinal study, factors that varied with time and changes in medical policies could not be controlled.

## 5. Conclusions

As a possible predictor, a history of IDA is a significant and independent risk factor for osteoporosis. Appropriate information and suggestions should be provided to people at-risk after confirmation of IDA to facilitate earlier medical management or specialty referral. Counseling and management can be considered for affected patients to improve symptoms resulting from osteoporosis, such as backache and joint pain, and to prevent known complications, such as fractures.

## Figures and Tables

**Table 1:** Characteristics of individuals in the case and control groups.

	Case Group (*n* = 35,751)		Control Group (*n* = 178,755)		OR	95% CI	*p*-Value
	No.	%	No.	%			
Age							
Age at IDA diagnosis (year-old))	43.65 (13.75)		43.65 (13.75)				1.00
Age at osteoporosis diagnosis (year-old)	59.13 (11.08)		62.45 (10.88)				<0.001
Gender							1.00
Female	29,017	81.16	145,085	81.16			
Male	6734	18.84	33,670	18.84			
Occupation							<0.001
Blue collar	11,113	31.08	50,900	28.47			
White collar	12,953	36.23	68,337	38.23			
Retired and others	11,685	32.68	59,518	33.30			
Urbanization							<0.001
Urban	21,504	60.15	113,705	63.61			
Suburban	10,803	30.22	51,025	28.54			
Rural	3444	9.63	14,025	7.85			
Insurable wage							<0.001
<20,000 NTD/month	7547	21.11	40,732	22.79			
20,001–40,000 NTD/month	15,766	44.10	75,009	41.96			
>40,000 NTD/ month	5114	14.30	26,249	14.68			
Retired and others	7324	20.49	36,765	20.57			
Co-morbidities							
Previous fractures	223	0.62	611	0.37	1.69	1.45–1.97	<0.001
Hypertension	7879	22.04	29,117	16.29	1.45	1.41–1.49	<0.001
Diabetes mellitus	5533	15.48	17,354	9.71	1.70	1.65–1.76	<0.001
Dyslipidemia	7685	21.50	25,815	14.44	1.62	1.58–1.67	<0.001
Congestive heart failure	977	2.73	1906	1.07	2.61	2.41–2.82	<0.001
Coronary artery disease	1628	4.55	5045	2.82	1.64	1.55–1.74	<0.001
Cerebrovascular disease	2856	7.99	8974	5.02	1.64	1.57–1.72	<0.001
Chronic pulmonary disease	9505	26.59	36,411	20.37	1.42	1.38–1.45	<0.001
Disorders of thyroid gland	3348	9.36	11,034	6.17	1.57	1.51–1.64	<0.001
Disorders of parathyroid gland	124	0.35	309	0.17	2.01	1.63–2.48	<0.001
Disorders of adrenal glands	244	0.68	689	0.39	1.78	1.53–2.06	<0.001
End-stage renal disease	1424	3.98	1799	1.01	4.08	3.80–4.38	<0.001
Liver disease	1036	2.90	1265	0.71	4.19	3.85–4.55	<0.001
Digestive ulcer or hemorrhage	13,437	37.58	45,216	25.29	1.78	1.77–1.82	<0.001
Menopause (female)	4138	11.57	16,903	9.46	1.25	1.21–1.30	<0.001
Disorders of menstruation (female)	17,291	48.37	63,507	35.53	1.70	1.66–1.74	<0.001

Case group: patients diagnosed with IDA; Control group: individuals without IDA who were matched to the case group by age and gender. Data are expressed as the number (%) or mean ± standard deviation, as appropriate.

**Table 2:** Hazards ratios of subsequent osteoporosis in patients diagnosed with IDA.

	Case Group (*n* = 35,751)		Control Group (*n* = 178,755)		Crude HR (95% CI)	Adjusted HR ^1^ (95% CI)	*p*-Value
	No.	%	No.	%			
**No osteoporosis**	34,940	97.73	176,389	98.68	1.74	1.81	<0.001
**Osteoporosis**	811	2.27	2366	1.32	(1.61–1.88)	(1.67–1.97)	

^1^ Adjusted for age, gender, occupation, urbanization, insurable wage, and co-morbidities.

**Table 3:** Risks for subsequent osteoporosis in patients diagnosed with IDA, stratified by gender.

	Case Group (*n* = 35,751)		Control Group (*n* = 178,755)		Crude HR (95% CI)	Adjusted HR ^1^ (95% CI)	*p*-Value
	No.	%	No.	%			
Male (*n* = 40,404)							
No osteoporosis	6662	98.93	33,493	99.47	2.08	1.99	<0.001
Osteoporosis	72	1.07	177	0.53	(1.58–2.73)	(1.50–2.65)	
Female (*n* = 174,102)							
No osteoporosis	28,278	97.45	142,896	98.49	1.71	1.80	<0.001
Osteoporosis	739	2.55	2189	1.51	(1.57–1.86)	(1.65–1.96)	

^1^ Adjusted for age, gender, occupation, urbanization, insurable wage, and co-morbidities.

**Table 4:** Risks for subsequent osteoporosis in case sub-groups according to treatment.

Intervention	Control Group (*n* = 178,755)		IDA Patients without Any Intervention or Oral Iron Therapy Alone (*n* = 26,263)			IDA Patients with Intravenous Ferrum Use (*n* = 4638)			IDA Patients with a Blood Transfusion (*n* = 4850)		
	Total No.	%	Total No.	%	*p*-Value	Total No.	%	*p*-Value	Total No.	%	*p*-Value
No osteoporosis	176,389	98.68	25,693	97.83		4501	97.05		4746	97.86	
Osteoporosis	2366	1.32	570	2.17	<0.0001	137	2.95	<0.0001	104	2.14	<0.0001
Crude HR (95% CI)	1.00		1.679		<0.0001	1.889		<0.0001	1.909		<0.0001
			(1.532–1.840)			(1.590–2.244)			(1.569–2.324)		
Adjusted HR ^1^ (95% CI)	1.00		1.800		<0.0001	2.206		<0.0001	1.468		0.0002
			(1.639–1.976)			(1.854–2.626)			(1.200–1.796)		

^1^ Adjusted for age, gender, occupation, urbanization, insurable wage, and co-morbidities.
